# Six years survival on imatinib with no disease progression after diagnosis of metastatic duodenal gastrointestinal stromal tumour: A case report

**DOI:** 10.1186/1752-1947-2-110

**Published:** 2008-04-18

**Authors:** Sayantan Bhattacharya, Amit Kumar Choudhury, Srinivasan Ravi, John Morrissey, George Mathew

**Affiliations:** 1 Department of General Surgery, George Eliot Hospital, College Street, Nuneaton, Warwickshire CV10 7DJ, UK; 2 Department of General Surgery, Victoria Hospital, Whinney Heys Road, Blackpool, Lancashire FY3 8NR, UK; 3 Institute of Clinical Sciences, Warwick Medical School, Coventry CV4 7AL, UK

## Abstract

**Introduction:**

A duodenal Gastrointestinal Stromal Tumour (GIST) is a rare finding and until recently advanced disease had a poor prognosis. A PubMed search revealed no reports of more than five years survival of inoperable GIST on chemotherapy with WHO performance status zero.

**Case Presentation:**

A 68 year old female was diagnosed with unresectable GIST in the duodenum with metastasis to liver, pancreas and omentum in November 2001. She was commenced on imatinib mesylate (Glivec) chemotherapy. This case report was prepared from the medical records and radiology reports. She had good tolerance with stable disease. After six years her CT scan showed no disease progression and her WHO performance status was zero.

**Conclusion:**

This report supports the view that imatinib is a safe and effective drug in controlling disease progression in advanced metastatic GIST and plays an important role in improving the patient's quality of life.

## Introduction

A duodenal tumour, especially a gastrointestinal stromal tumour (GIST), is a rare finding and until recently advanced disease had a poor prognosis. A PubMed search revealed only a few reports of prolongation of the lives of patients with advanced duodenal GIST by treatment with imatinib. The maximum length of follow up was 29 months [1] from diagnosis. A recent study suggested that the prognosis for unresectable and/or metastatic GIST is poor with few if any patients surviving more than five years [2].

## Case Presentation

A previously fit and well, 68-year-old female presented in November 2001 with recent onset of epigastric discomfort, significant loss of appetite and melaena. She had also lost 5 kilos in weight over a three-week period. Over the previous few weeks, she was mostly confined to bed, as she was extremely lethargic.

**Figure 1:**
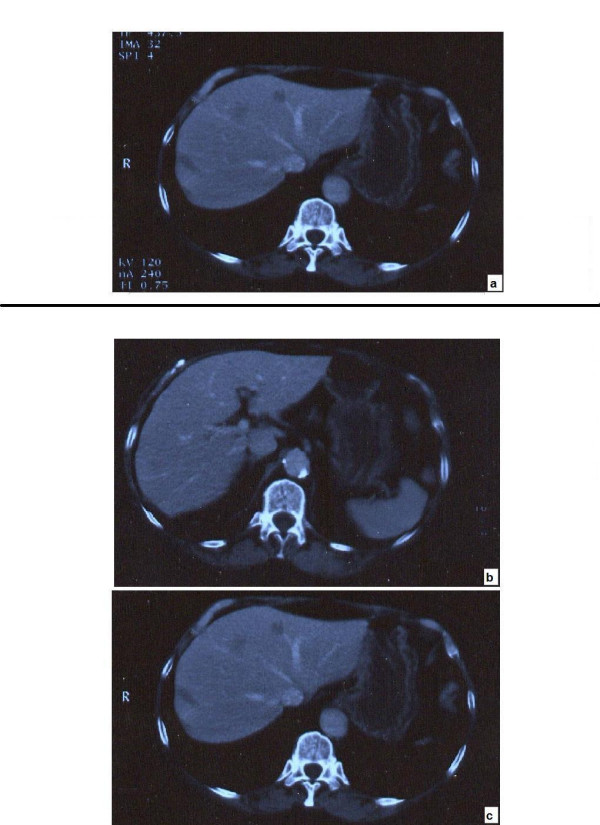
**Microscopy and Endoscopy pictures at diagnosis in November 2001**. **a**: shows pictures of H and E staining, **b**: KIT positive staining **c**: Endoscopic findings.

**Figure 2:**
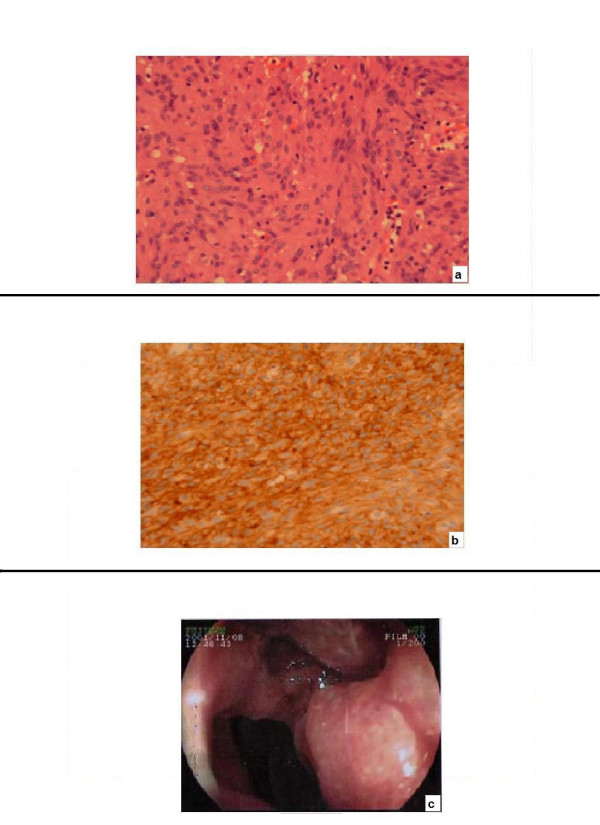
**CT scan images**. **a**: at diagnosis (November 2001) **b and c**: at the last follow up (in December 2007).

Upper gastrointestinal endoscopy (Figure 1c) revealed a pre-ampullary tumour in the second part of the duodenum. Histology confirmed the tumour to be a GIST with more than 10 mitotic activities per 50 high power fields (HPF) (Figure 1a). The tumour was found to be positive for a deletion mutation in exon-11 (of the c-KIT exon) (Figure 1b). Computerised tomography (CT) of the abdomen showed multiple metastatic deposits in the liver (Figure 2a). At laparotomy the tumour was found to involve the liver, pancreas and omentum. A hard lump in the right paracolic gutter was inseparable from the right kidney. Owing to the extensive nature of the disease the abdomen was closed without any resection.

Oncology assessment recorded a WHO performance status of three (see Additional file 1). She was commenced on Imatinib Mesylate (Glivec) 400 mg once daily. By September 2002 her WHO performance status was zero. She had mild nausea but her appetite was normal and she had no urinary or bowel symptoms. Full blood counts and renal function tests were normal. Her liver function tests were abnormal even before treatment, probably a result of hepatic metastases, and this persisted.

After 28 months of treatment she developed minor side effects including infrequent diarrhoea and watery painful eyes. Her regime was modified to four weeks on treatment with an off period for the next two weeks and this improved her symptoms. The daily dose remained at 400 mg. At her most recent review in December 2007 she remained symptomatically well. She was mobile and had no difficulties in performing her daily activities. She was followed up with a CT scan (Figure 2b and 2c) but she refused any further endoscopic investigations.

## Discussion

GIST is a rare form of sarcoma arising either from the interstitial cells of cajal (ICCs) or from less differentiated stem cells. The ICCs are located in the muscle layer of the gastrointestinal tract. GIST can occur anywhere but mainly affects the stomach (60%), jejunum and ileum (30%), duodenum (4–5%), rectum (4%), colon and appendix (1–2%) and oesophagus (<1%) [3]. Rarely extra-gastrointestinal GIST occurs in the vicinity of the stomach or intestines [3]. The overall incidence has been estimated at 10–20 per million.

There has been extensive study of GIST to determine whether tumour size, mitotic activity and genetic characteristics predict disease progression. GISTs more than 5 cm in size, independent of mitotic rate, have a moderate risk for metastases, and all tumours with less than 5 mitoses per 50 HPFs have a high risk for metastases. Miettinen and Lasota reasoned that when data are analysed by regression models tumour size may appear to show greater predictive value because mitotic count reaches a 'saturation point' when counts exceed 10 per 50 HPFs [3].

Bearzi et al [4] reviewed 158 cases of GIST. Only 12% of patients with a mitotic count over 10 per 50 HPFs remained disease-free after surgery, and all patients with a mitotic count over 20 per 50 HPFs experienced recurrence. They argued that if the combination of size and mitotic count place a tumour in the high-risk category, then the mitotic count might be the more indicative variable.

Recent papers suggest a mutation found in a GIST may predict its likelihood of recurrence. Emile et al [5] and Martin et al [6] identified specific mutations within c-KIT exon 11 which were associated with metastasis and poor prognosis. Several papers have found that patients with deletions in exon-11 rather than substitutions or duplications have a greater probability of recurrence or metastasis than other GIST patients [7,8].

If these results are confirmed and expanded they could provide a rationale for identifying patients who need closer monitoring or perhaps adjuvant chemotherapy post-surgery to prevent recurrence rather than after the appearance of metastasis in line with guidance by the UK National Institute of Clinical Excellence (NICE; see Additional file 2).

Cell proliferation in a GIST is a result of the activation of growth factor receptors. KIT and platelet derived growth factor receptor alpha (PDGFRA) tyrosine kinases [9] are normally present on the ICCs. When the genes of these receptor cells are mutated activation take place without stimulation by the respective ligands (constitutive activation). This causes tumour growth.

Imatinib Mesylate (Glivec) is the first effective treatment for GIST. Imatinib binds to the intracellular activation pockets of the KIT and PDGFRA receptors in their inactive position, blocking their binding to ATP and preventing growth signals being sent, which stops disease progression. Drug response is related to the type of mutation of the c-KIT exon. Patients with the more common exon-11 mutation are most sensitive to imatinib [10] whereas patients with the exon-9-mutation, mostly found in those with small intestinal GIST, are the least sensitive [11].

The most commonly reported side effects of imatinib are nausea, diarrhoea, periorbital oedema, muscle cramps, fatigue, rash and headache. The most common serious adverse events are unspecified haemorrhage and neutropenia, each occurring in approximately 5% of patients (see Section 4.1.9 in [2]).

In our patient, imatinib was extremely effective in controlling the disease process. This was most likely a result of the type of mutation (exon-11) that was present in this tumour. The WHO performance status at diagnosis was three. Since September 2002, her WHO performance status has remained at zero. She had suffered from minor side effects of the drug like diarrhoea and watery painful eyes. She was reviewed in the oncology clinic and the change of regime to four weeks on and two weeks off the drug relieved these side effects.

## Conclusion

There have been articles published on the effectiveness of imatinib but very few have shown a six-year disease-controlled survival with a good quality of life when extensive spread of the disease was apparent at diagnosis. NICE [2] guidance (see Additional file 2) suggests imatinib should only be used in patients with established metastases, unresectable disease or residual disease following surgery. The effectiveness of this drug is dependent on the type of mutation of the c-KIT exon. Knowledge of this mutation would thus help in assessing the prognosis of treatment. We believe our report supports the view that imatinib is a safe and effective drug in controlling disease progression in advanced metastatic GIST and plays an important role in improving the patient's quality of life.

## List of abbreviations

GIST: Gastrointestinal Stromal Tumour, ICC: Interstitial Cells of Cajal, WHO: World Health Organization, HPF: High Power Field, ATP: Adenine Triphosphate

## Competing interests

The authors declare that they have no competing interests.

## Authors' contributions

SB and AKC were actively involved in the follow-up of the patient, obtained the consent from the patient and prepared the final version of manuscript. SR was the consultant colorectal and GI surgeon under whom the concerned patient was admitted and managed. He has important contributions in preparation of the final version of the manuscript. JM and GM had separately revised the final version of the manuscript before the last submission and had important contribution to the discussion section of the manuscript. All the authors have read and approved the final version of the manuscript.

## Consent

Written informed consent was obtained from the patient for publication of this case report. A copy of the written consent is available for review by the Editor-in-Chief of this journal.

## Supplementary Material

Additional file 1 WHO scale for assessing the performance status of the patient. The table describes the WHO performance status in patients. Click here for file

Additional file 2 NICE guidelines on Imatinib therapy in GIST. The table describes the NICE guidelines for using Imatinib mesylate in GIST patients. Click here for file
