# Supplementary figures and images for: Six years survival on imatinib with no disease progression after diagnosis of metastatic duodenal gastrointestinal stromal tumour: A case report
Source: J Med Case Rep. 2008 Apr 18;2:110. doi: 10.1186/1752-1947-2-110 (PMC2386476; doi:10.1186/1752-1947-2-110)

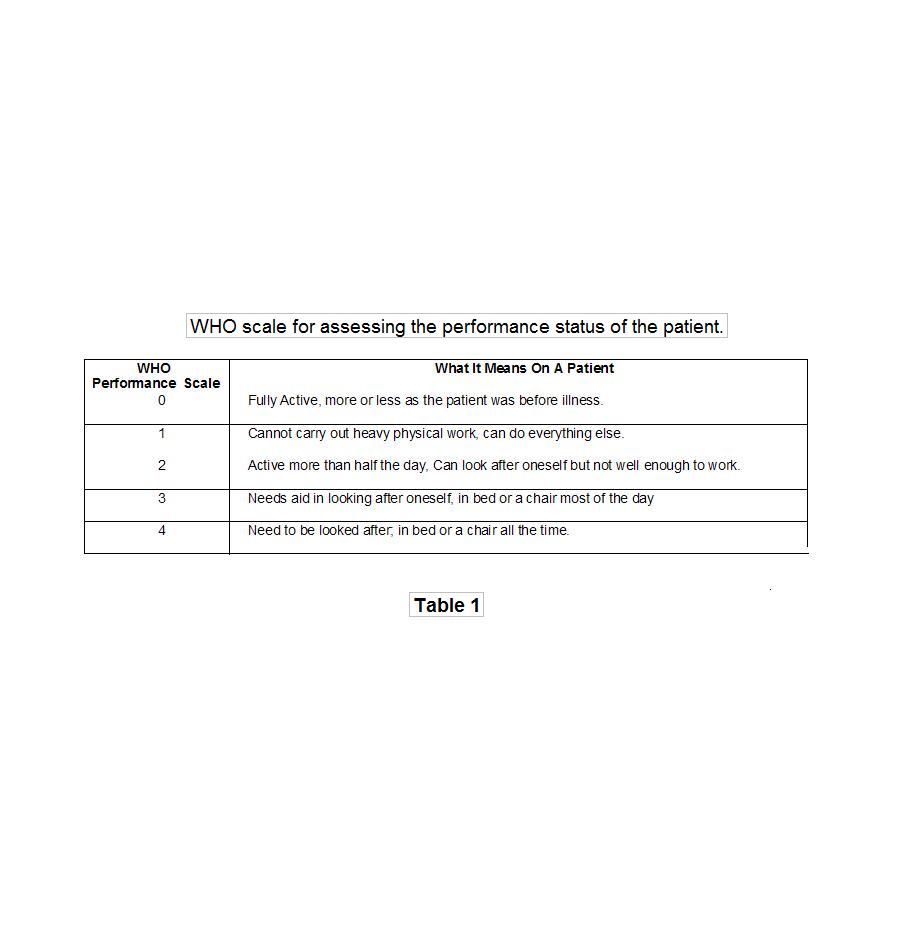

Supplement: Additional file 1 — WHO scale for assessing the performance status of the patient. The table describes the WHO performance status in patients. [file 1752-1947-2-110-S1.jpeg]

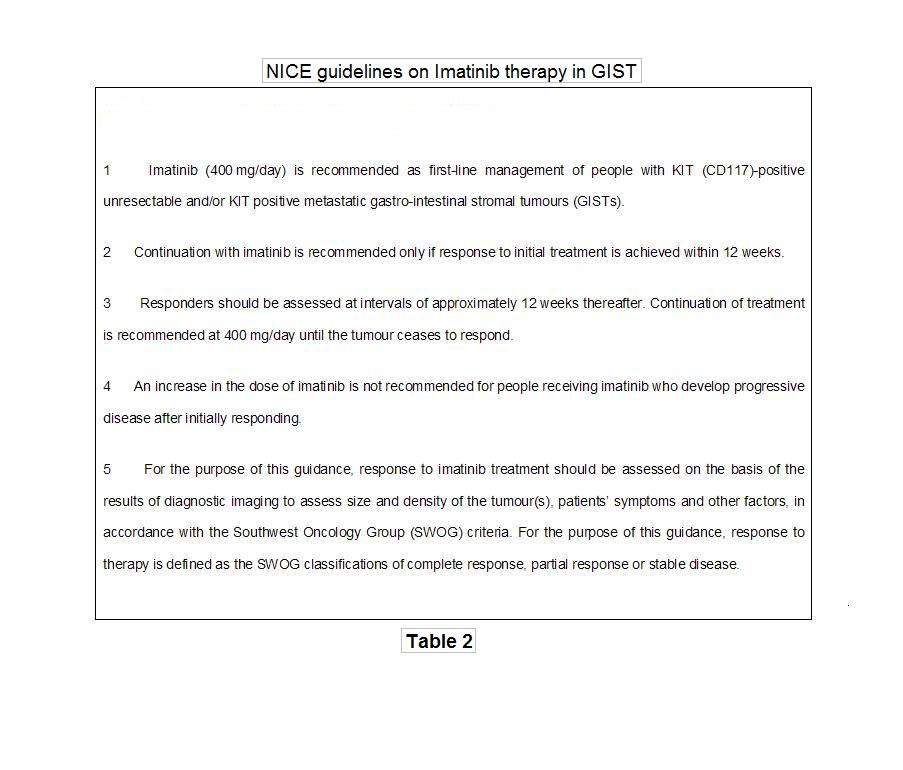

Supplement: Additional file 2 — NICE guidelines on Imatinib therapy in GIST. The table describes the NICE guidelines for using Imatinib mesylate in GIST patients. [file 1752-1947-2-110-S2.jpeg]
